# The Paf1 complex promotes displacement of histones upon rapid induction of transcription by RNA polymerase II

**DOI:** 10.1186/1471-2199-9-4

**Published:** 2008-01-14

**Authors:** Heather A Marton, Stephen Desiderio

**Affiliations:** 1 Department of Molecular Biology and Genetics and Program in Immunology, Institute for Cell Engineering, The Johns Hopkins University School of Medicine, 733 North Broadway, Baltimore, Maryland 21205, USA

## Abstract

**Background:**

The yeast Paf1 protein complex is required for efficient transcription elongation by RNA polymerase II (RNA pol II), but the precise role of the complex has been unclear.

**Results:**

Here we show that depletion of the Ctr9 or Paf1 component of the Paf1 complex delays the loss of histones from the *GAL1 *gene upon induction. This delay in histone removal is accompanied by a decrease in association of RNA pol II with *GAL1 *and altered distribution of the polymerase along the locus.

**Conclusion:**

These observations may explain why initial induction of *GAL *transcripts is reduced in Ctr9- or Paf1-deficient cells, and is consistent with a model suggesting that the Paf1 complex and the histone modifications that it mediates increase efficiency of transcriptional elongation by promoting nucleosomal destabilization and histone removal.

## Background

Transcription by RNA polymerase (pol) II is subject to complex regulation at the level of chromatin modification and assembly. Several classes of chromatin modifying proteins have been identified; these contribute to gene activation by promoting chromatin decondensation, thereby allowing access of RNA pol II to regulatory sequences and facilitating movement of RNA pol II and associated factors through transcription units. ATP-dependent remodeling factors, including SWI/SNF and NURF, disrupt nucleosome structure and increase DNA accessibility [1]. Histone acetyltransferases (GNAT and MYST families) promote chromatin opening, nucleosome fluidity and protein interactions by acetylating histones at specific lysine residues [2]. Histone methyltransferases (CARM1 and MLL-families) modify histones at lysine and arginine; methylation of particular residues, for example, on histone H3 at K4 and K36, is linked to active transcription and may mark recently transcribed regions [3].

Efficient transcriptional elongation through chromatin requires additional factors, including FACT, Spt6 and Asf1, which are required for histone eviction and histone redeposition [4-7]. Mutation of each of these factors is associated with inappropriate nucleosomal positioning and activation of cryptic promoters within coding regions [8-10]. FACT and Asf1 may facilitate elongation by stimulating the eviction of H2A/H2B or H3/H4 dimers, respectively, from chromatin.

The Paf1 complex, which was first identified in yeast as a functional and physical cluster of proteins including Paf1, Ctr9, Cdc73, Rtf1 and Leo1 [11-13], is also required for efficient transcriptional elongation through chromatin [14,15]. The Paf1 complex is associated with transcriptionally active RNA pol II and interacts with the transcription elongation factors Spt4, Spt5, FACT and TFIIS [11,13,16]. Paf1 and Rtf1 are required for monoubiquitylation of histone H2B by Rad6-Bre1 at K123 [17,18]. This modification, in turn, is essential for the di- and tri-methylation of histone H3 by the Set1 and Dot1 methyltransferases at K4 and K79, respectively [19-21]. Moreover, Paf1 and Rtf1 promote the recruitment of Set1 and Set2, a histone H3 K36 methyltransferase, to RNA pol II at sites of active transcription [22,23].

In this communication we show that depletion of the Ctr9 or Paf1 component of the Paf1 complex is associated with delayed loss of histones from the *GAL1 *gene upon induction, decreased association of RNA pol II with *GAL1 *and altered distribution of the polymerase along the *GAL1 *locus. These observations are consistent with the interpretation that the Paf1 complex enhances transcriptional elongation by promoting nucleosomal destabilization and histone removal.

## Results

### Correlation of transciption initiation frequency with occupancy by Ctr9 and Paf1

**Figure 1:**
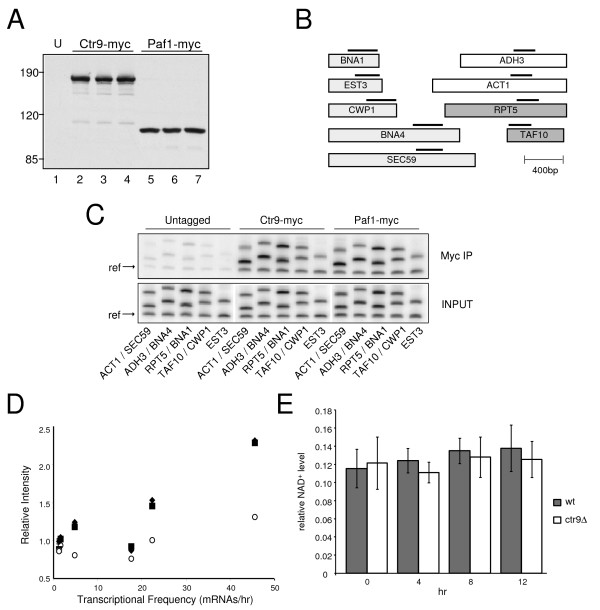
**Gene occupancy by Ctr9 and Paf1**. (*A*) Expression of endogenous, c-myc-tagged Ctr9 and Paf1 proteins. Protein from three Ctr9-myc-expressing isolates (HM198), three Paf1-myc-expressing isolates (HM199) or the untagged HM177 parental strain (U) were fractionated by SDS-PAGE. Protein was detected by immunoblotting with the 9E10 antibody. (*B*) Locations of gene-specific PCR probes. Each ORF is represented by a box. Light gray, genes whose expression decreased upon removal of Ctr9; dark gray, genes whose expression increased; white, genes whose expression remained unchanged. The positions and relative sizes of PCR products are indicated by dark bars above. (*C*) Assays of gene occupancy by ChIP. Association of Ctr9 and Paf1 with the regions indicated in (*B*) was monitored in cells maintained in YPD medium at 30°C. Upper panel, amplification of anti-myc immunoprecipitates; lower panel, amplification of input chromatin. The middle and upper bands in each lane represent amplified segments of the ORFs indicated (middle/upper) at bottom. The lowest band in each lane is an amplified fragment from the promoter region of *ARN1*, which was used as an internal reference [16]. (*D*) Correlation of transcriptional initiation frequency with gene occupancy by Ctr9 and Paf1. The relative signal intensities for each of six gene fragments associated with Ctr9-myc and Paf1-myc are plotted as a function of their transcriptional initiation frequencies as estimated in reference [25]. Diamonds, Paf1-myc; squares, Ctr9-myc; circles, untagged control. (*E*) Effect of acute Ctr9 depletion on intracellular NAD^+ ^levels. Wild-type (HM167) and *ctr9Δ *(HM158) strains containing the Ctr9 expression plasmid pgCTR9M2 were initially grown in SC -leu + 2% galactose. Cells were then transferred to SC -leu + 2% glucose and maintained at 30°C. Relative intracellular levels of NAD^+ ^were measured at 0, 4, 8 and 12 hours after switch to glucose. NAD^+ ^levels are expressed on the Y axis as absorbance at 340 nm. Values represent mean and standard deviation (n = 3).

We began by examining association of Ctr9 and Paf1 with genes whose expression increased, decreased or remained unchanged upon depletion of Ctr9 or Paf1, as determined by microarray analysis ([24] and data not shown). Endogenous Ctr9 and Paf1 were each tagged at the carboxyl terminus with multiple c-myc epitopes; expression of tagged proteins was confirmed by immunoblotting (Fig. 1A). Association of tagged Paf1 and Ctr9 with specific open reading frames (ORFs) (Fig. 1B) was assayed by chromatin immunoprecipitation (ChIP).

Contrary to expectation, occupancy of an ORF by either protein was not correlated with its change in expression upon depletion of Ctr9 (Fig. 1C). For example, association of Ctr9 and Paf1 was greatest with *ACT1 *and *ADH3*, two genes whose expression was unchanged in the *ctr9Δ *strain, while association of these proteins was lowest with *EST3*, whose expression decreased in response to Ctr9 depletion. Rather, the occupancy of a specific ORF by Ctr9 or Paf1 was positively correlated with the frequency of transcriptional initiation at that locus, as estimated by Young and coworkers ([25] and Fig. 1D). These findings suggest that the occupancy of a locus by the Paf1 complex is positively correlated with its occupancy by RNA pol II.

We next sought a common feature of transcriptional regulation among the genes in the downregulated set. A subset of *BNA *genes, which function in NAD^+ ^biosynthesis, is down regulated in *ctr9Δ *cells (data not shown). These genes are coordinately repressed by the Sir2 homologue Hst1, a sensor of intracellular NAD^+ ^[26]. This observation suggested that Ctr9 might function, directly or indirectly, in relief of repression by Hst1. Although the down regulation of *BNA *genes observed in *ctr9Δ *cells might have resulted from an increase in intracellular NAD^+^, no significant change in the amount of intracellular NAD^+ ^was found (Fig 1E). Thus, the effect of Ctr9 depletion on expression of NAD^+ ^biosynthetic genes was not likely to be mediated by NAD^+ ^itself, but rather through a more direct effect on transcription. Together, these results raised the possibility that deleterious effects of Paf1 or Ctr9 depletion on transcription might be most readily observed at loci that are subject to fluctuation between active and silent states or that are capable of rapid induction.

### Impaired transcription of telomeric genes upon loss of Ctr9

**Figure 2:**
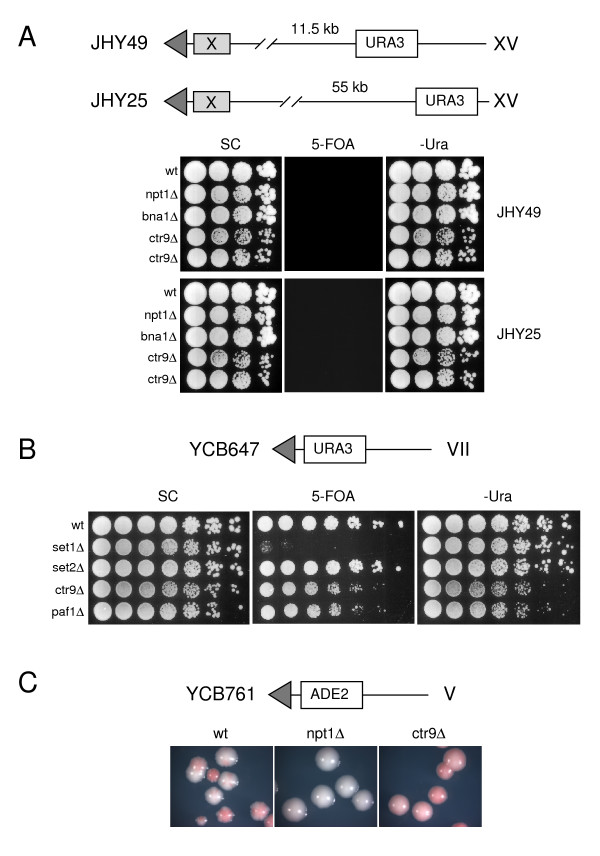
**Position-dependent effects of *CTR9 *deletion on transcription and silencing**. (*A*) The wild-type strains JHY49 and JHY25 each contain a single copy of *URA3 *integrated 11.5 Kb and 55 Kb, respectively, downstream of the natural left telomere of chromosome XV, as diagrammed above. Into these strains were introduced *npt1Δ *(HM193, HM190), *bna1Δ *(HM192, HM189) and *ctr9Δ *(HM191, HM188) deletions. Cells were diluted serially 10-fold and plated on SC, 5FOA or SC -ura as indicated. (*B*) The wild-type YCB647 strain carries a single copy of *URA3 *integrated into the left telomere of chromosome VII, as diagrammed above. Into this strain, *set1Δ *(HM206), *set2Δ *(HM207), *ctr9Δ *(HM180) or *paf1Δ *(HM205) deletions were introduced. Cells were diluted serially 5-fold and plated on SC, 5FOA, or SC -ura as indicated. (*C*) The wild-type YCB761 strain contains a single copy of *ADE2 *integrated into the right telomere of chromosome V as shown above. This and derivatives carrying *npt1Δ *(JS692) or *ctr9Δ *(HM182) deletions were plated on SC containing limiting adenine.

To test whether the Paf1 complex contributes to the establishment of transcriptionally permissive chromatin, we examined the effect of Ctr9 or Paf1 depletion on transcription of *URA3 *in its natural setting and at three other sites. Expression of *URA3 *from its natural locus was unaffected by loss of Ctr9 (data not shown). Additionally, expression of *URA3 *incorporated into sites not subject to telomeric silencing, either 11.5 or 55 kb downstream of the natural telomere on chromosome XV [27] was unperturbed (Fig. 2A). Wild-type and *ctr9Δ *cells, as well as the control strains *npt1Δ *and *bna1Δ*, which carry deletions in genes implicated in maintenance of silencing, grew similarly on plates lacking uracil, while growth was completely inhibited by 5-FOA. In contrast, when *URA3 *was integrated adjacent to an artificial telomere on chromosome VII [28], growth of Ctr9- or Paf1-deficient cells was inhibited on medium lacking uracil (Fig. 2B), indicating a context-dependent effect on *URA3 *expression. The growth of these cells was also impaired in the presence of 5-FOA, consistent with impairment of telomeric silencing, as previously noted [17,29], although the effects of *CTR9 *or *PAF1 *deletion were less severe than observed for a *set1Δ *strain (Fig. 2B).

One interpretation of these results is that deletion of *CTR9 *or *PAF1 *impairs gene expression in the setting of an artificial telomere, while simultaneously reducing silencing. This interpretation was supported by examining expression of *ADE2*, integrated adjacent to an artificial telomere on chromosome V [28], in the presence or absence of Ctr9 (Fig. 2C). In this assay, cells expressing *ADE2 *appear white, while cells in which *ADE2 *is silenced appear red. On a wild-type background, as expected, sporadic loss of telomeric silencing at the *ADE2 *locus was associated with the appearance of red and white sectored colonies (Fig. 2C). Loss of *NPT1*, which encodes a component of the NAD^+ ^salvage pathway, completely abolished silencing; as a result all colonies were white. On a *ctr9Δ *background, in contrast, colonies exhibited a uniform pink colour (Fig. 2C). This phenotype is consistent with expression of *ADE2 *at an intermediate level in most or all cells, as would be expected if loss of Ctr9 were to relieve telomeric silencing and at the same time to reduce transcriptional efficiency. These observations demonstrate position-dependent impairment of gene expression in the absence of Ctr9 and Paf1, and once again suggest that the Paf1 complex facilitates transcription through regions of chromatin that are undergoing a transition from inactive to active states. We proceeded to test this directly by examining the effects of Ctr9 and Paf1 depletion on induction of repressed loci.

### Impaired induction of *GAL *genes in the absence of Ctr9 or Paf1

Ctr9 and Paf1 are required for monoubiquitylation of H2B at K123 [17,18], a process which in turn is necessary for efficient expression of *GAL *genes [30-32]. We examined the effects of Ctr9 or Paf1 depletion on induction of transcription at *GAL *loci. Strains were grown with raffinose as the sole carbon source before induction with galactose; this allowed us to bypass the process of derepression and focus solely on transcriptional activation.

**Figure 3:**
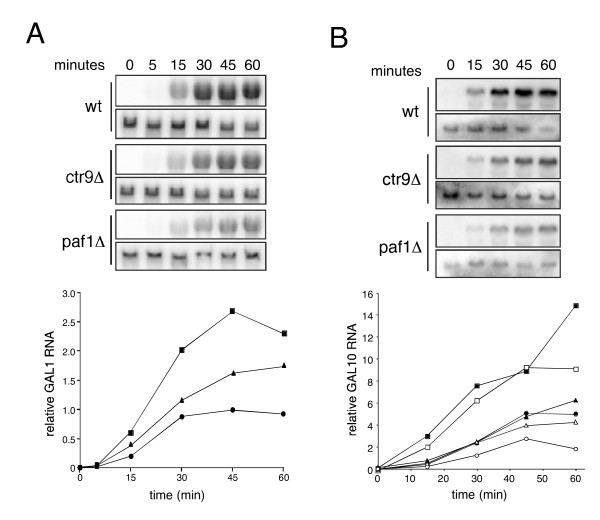
**Impaired induction of *GAL *loci in cells deficient in Paf1 or Ctr9**. (*A*) Induction of *GAL1 *transcripts. Wild-type (HM200), *ctr9Δ *(HM201) and *paf1Δ *(HM202) strains were grown at 30°C in YEP supplemented with 2% raffinose. Galactose was added to a final concentration of 2%. RNA was isolated at 0, 5, 15, 30, 45 and 60 min after addition of galactose and *GAL1 *(upper panels) and *ACT1 *(lower panels) transcripts were detected by northern blotting (above). The *GAL1 *signal intensities were normalized to those of *ACT1 *and plotted as a function of time (below). Filled squares, wild-type; filled triangles, *ctr9Δ *; filled circles, *paf1Δ*. (*B*) Induction of *GAL10 *transcripts. Isolates of wild-type (HM200), *ctr9Δ *(HM201) and *paf1Δ *(HM202) were grown at 24°C in media indicated above. Galactose was added as described and RNA was isolated at 0, 15, 30, 45 and 60 min after addition. *GAL10 *(upper panels) and *ACT1 *(lower panels) transcripts were detected by northern blotting (above). *GAL10 *signal intensities were normalized as in (*A*) and plotted together with data obtained from an independent experiment using a second set of wild-type, *ctr9Δ *and *paf1Δ *isolates. Filled and open squares, wild-type; filled and open triangles, *ctr9Δ*; filled and open circles, *paf1Δ*.

Induction of *GAL1 *RNA was impaired in both the *ctr9Δ *and *paf1Δ *strains, relative to wild-type (Fig. 3A). Deletion of *CTR9 *or *PAF1 *was associated with a 2- to 3-fold reduction in *GAL1 *RNA at 30 min after induction by galactose. We asked whether this decrease in RNA accumulation was accompanied by a change in transcriptional kinetics by assaying induction of *GAL10 *by galactose at 24°C. By inducing expression at a reduced temperature we hoped to unmask differences in the rate of transcript accumulation that might have been less pronounced at 30°C. We also used additional isolates of the wild-type, *ctr9Δ *and *paf1Δ *strains to militate against potential effects of secondary mutations cause by loss of Ctr9 or Paf1. Deletion of *CTR9 *or *PAF1 *had substantial effects on the initial accumulation of *GAL10 *transcripts, reducing RNA levels by about 5 fold at 15 min; by 30 min the difference in transcript levels between wild-type and the mutants was 3 fold and by 45 min this was reduced to only a 2-fold difference (Fig. 3B). We obtained similar results for *GAL7 *(data not shown). These results indicated that loss of the Paf1 complex is associated with delayed induction of *GAL *loci, consistent with preferential impairment of early transcription in the absence of the Paf1 complex, as has been described [14].

### Loss of Ctr9 or Paf1 impairs displacement of histones upon *GAL1 *induction

**Figure 4:**
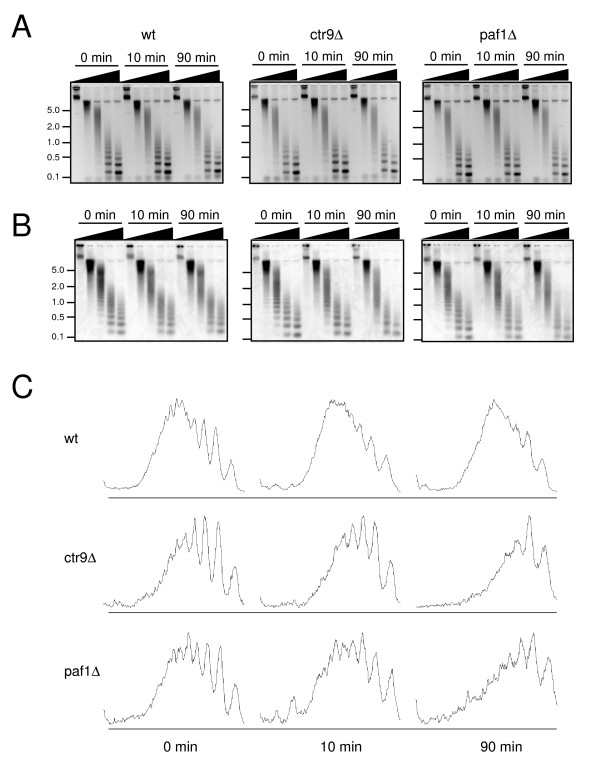
**Deletion of *CTR9 *or *PAF1 *impairs displacement of histones from the *GAL1 *locus upon induction**. Wild-type (HM200), *ctr9Δ *(HM201) and *paf1Δ *(HM202) strains were grown at 24°C in YEP supplemented with 2% raffinose. Galactose was added to a final concentration of 2%. Nuclei were isolated at 0, 10 and 90 min after induction and digested with 0, 7, 15, 50 or 100 U of micrococcal nuclease. (*A*) Micrococcal nuclease digests of nuclei from wild-type (left), *ctr9Δ *(middle) and *paf1Δ *(right) strains were deproteinized and fractionated by agarose gel electrophoresis; DNA was detected by ethidium bromide staining. Inverse images of the stained gels are shown. Positions of markers and their sizes (in kb) are indicated at left. (*B*) Distribution of micrococcal nuclease digestion products at the *GAL1 *locus. DNA from gels in (*A*) was transferred to nylon and hybridized to a probe representing basepairs +422 to +925 of the *GAL1 *gene. Signal was detected by phosphorimaging. (*C*) Distributions of micrococcal nuclease products from the 50 U micrococcal nuclease reactions in (*B*) were quantified by densitometry for each strain at each time point. The signal intensities are plotted in arbitrary units.

Induction of transcription at *GAL *loci is accompanied by loss of histones at promoter and coding regions [33-35]. We assessed the effects of *CTR9 *or *PAF1 *deletion on eviction of histones from the *GAL1 *locus upon induction. We first used micrococcal nuclease digestion of chromatin to examine nucleosomal occupancy and spacing before and after induction by galactose. Examination of bulk chromatin revealed no effect of *CTR9 *or *PAF1 *deletion on the general deposition or spacing of histones (Fig. 4A). Specific interrogation of the *GAL1 *locus, however, indicated that loss of either Ctr9 or Paf1 was associated with delayed displacement of histones from the gene. At 0 min, a discrete distribution of nucleosomal digestion products was observed in all samples. In the wild-type sample, this discrete distribution was largely lost by 10 min after galactose induction, and this pattern persisted at 90 min after induction (Fig. 4B and 4C). This suggested that in the wild-type strain, nucleosomes had been lost or their phasing disrupted by 10 min after induction. In contrast, discrete nucleosomal laddering was still observed at 10 min after galactose induction in the *ctr9Δ *and *paf1Δ *strains (Fig. 4B and 4C). By 90 min, nucleosome occupancy or phasing had been lost in all strains. The difference between wild-type and *ctr9Δ *or *paf1Δ *strains with respect to the kinetics of phased nucleosome loss was consistent with the interpretation that the Paf1 complex is required for the timely removal of histones from the *GAL1 *gene, or their displacement over the locus, upon induction of transcription. To distinguish between these possibilities, we used ChIP to determine whether depletion of Ctr9 or Paf1 affects the association of histones and RNA pol II with the *GAL1 *locus upon activation.

**Figure 5:**
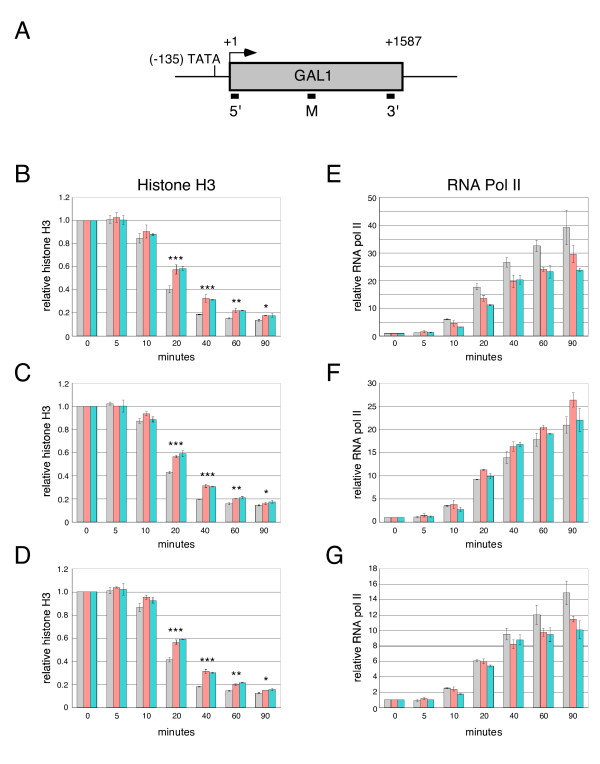
**Depletion of Ctr9 or Paf1 delays removal of histone H3 and alters the distribution of RNA pol II upon induction of *GAL1 *transcription**. (*A*) Schematic representation of the *GAL1 *locus. The TATA region, translation start site and ORF are indicated. Black bars below indicate positions of PCR probes used in ChIP assays. (*B – G*) Association of histone H3 (*B – D*) or RNA pol II (*E – G*) with 5' (*B, E*), middle (*C, F*) and 3' (*D, G*) regions of the *GAL1 *locus as a function of time after induction. Wild-type (HM200), *ctr9Δ *(HM201) and *paf1Δ *(HM202) strains were grown at 24°C in YEP supplemented with 2% raffinose. Galactose was added to final concentration of 2%. Samples were taken at 0, 5, 10, 20, 40, 60 and 90 min after induction. Association of histone H3 (*B – D*) and Rpb3 (*E – G*) with 5' (*B, E*), middle (*C, F*) and 3' (*D, G*) regions of *GAL1 *were assayed by ChIP and real-time PCR for wild-type (gray), *ctr9Δ *(peach) and *paf1Δ *(turquoise) strains. All samples were also assayed with primers specific for an intergenic region (*B – D*) or *FBA1 *(*E – G*) as standards for normalization of the *GAL1*-specific signals. The normalized association of H3 or Rpb3 with each region of *GAL1 *at 0 min was set to 1; all other values represent the fold difference relative to the 0 min sample. Each value is the average of two experiments; error bars correspond to the standard errors of the mean. Statistical significance of pairwise comparisons between wild-type and *ctr9Δ *or *paf1Δ *strains was determined using a two-tailed t-test. ***, P < 0.0005; **, P < 0.004; *, P < 0.04.

Association of histone H3 and RNA pol II with 5', middle and 3' regions of *GAL1 *(Fig. 5A) were examined in wild-type, *ctr9Δ *and *paf1Δ *strains maintained at 24°C before and at various times after induction by galactose. In wild-type cells, occupancy of *GAL1 *by histone H3 decreased sharply from 10 to 40 min after induction and remained low for the remainder of the time course (Fig. 5B – D). Deletion of either *CTR9 *or *PAF1 *impaired removal of histone H3 from the *GAL1 *locus (Fig. 5B – D). Before the 10 min time point, no statisically significant differences in histone H3 levels were detected among wild-type, *ctr9Δ *and *paf1Δ *samples. By 20 min, histone H3 occupancy was about 30% lower in wild-type cells than in the *ctr9Δ *and *paf1Δ *mutants and this difference increased to 40% by 40 min. These differences were determined to be highly significant (P < 0.0005). By 90 min after induction, depletion of histone H3 over the *GAL1 *locus in the *ctr9Δ *and *paf1Δ *cells began to approach that of wild-type, but nonetheless remained statistically higher (P < 0.04). These results suggest that the Paf1 complex promotes eviction of histones from the *GAL1 *locus during early stages of transcriptional induction. Moreover, these findings indicate that in the absence of the Paf1 complex, nucleosomes can be removed from the *GAL1 *locus upon activation, but with delayed kinetics relative to wild-type.

In the nuclease digestion experiments we had observed more unphased DNA fragments of intermediate size in the wild-type sample than in the *ctr9Δ *and *paf1Δ *strains at 90 min (Fig. 4C). While this in principal could have reflected greater occupancy of the *GAL1 *locus by unphased nucleosomes in wild-type cells, our observation that removal of histones from *GAL1 *is more rapid in the wild-type than in *ctr9Δ *or *paf1Δ *strains argues against this point.

We observed an inverse relationship between histone displacement and the association of RNA pol II with the *GAL1 *locus. In wild-type cells, association of RNA pol II with *GAL1 *increased rapidly after addition of galactose (Fig. 5E – G). This association was apparent at 10 min after induction and continued to increase throughout the time course. The amount of RNA pol II at the 5' region was consistently greater than at the middle or 3' region of the gene, although the kinetics of polymerase association with these regions paralleled the kinetics observed for the 5' region (Fig. 5E – G). These findings are consistent with a report describing kinetics of association of RNA pol II with a galactose-inducible, synthetic locus [34].

Loss of Ctr9 or Paf1 was associated with changes in the association of RNA pol II with *GAL1 *and its distribution over the locus following induction. While the confidence limits determined for these differences were not below the significance threshold, similar trends were observed for the *ctr9Δ *and *paf1Δ *mutants. As with wild-type cells, association of RNA pol II with the 5' region of *GAL1 *was detectable in *ctr9Δ *and *paf1Δ *cells at 10 min after addition of galactose, but the amount of association was reduced (Fig. 5E). Throughout the time course, the amount of RNA pol II over the 5' region was consistently lower in the *ctr9Δ *and *paf1Δ *strains than in wild-type. Interestingly, loss of either Ctr9 or Paf1 was associated with a relative increase in the amount of RNA pol II associated with the middle region of the *GAL1 *locus (Fig. 5F), despite the reduction in the amount of polymerase associated with the 5' region. Only small differences in polymerase occupancy of the 3' region of *GAL1 *were observed among the strains (Fig. 5G). Deletion of *CTR9 *or *PAF1 *had similar effects on RNA pol II occupancy as well as histone displacement at the *GAL10 *locus (data not shown). These findings suggest that upon induction of transcription at *GAL1 *and *GAL10 *loci, the Paf1 complex promotes histone displacement and more efficient transcriptional elongation. We considered the possiblity that inappropriate internal initiations in the *ctr9Δ *and *paf1Δ *strains might contribute to alterations in the distribution of RNA pol II, but this seems unlikely as we observed no truncated *GAL1 *or *GAL10 *transcripts in hybridization assays of mutant cells using double-stranded probes, which would have detected transcripts from either strand (Fig. 3).

## Discussion

It has been reported that the Paf1 complex associates with RNA pol II and that loss of the Ctr9 or Paf1 components of this complex impairs transcription of a subset of genes. We find that the amount of Paf1 associated with a particular gene is positively correlated with its rate of transcription initiation. The Paf1 complex supports specific histone modifications that are essential for maintenance of telomeric silencing, including monoubiquitylation of histone H2B and methylation of histone H3 at lysine 4. In agreement with previous reports, we detect a loss of telomeric silencing in *CTR9 *and *PAF1 *deletion mutants, but demonstrate in addition that the loss of these genes also impairs transcription of telomeric markers.

The Paf1 complex is likely required for efficient transcriptional elongation [15], but the underlying mechanism has remained unclear. A recent report indicates that Paf1 complex-dependent ubiquitylation of H2B is required for efficient transcription elongation *in vitro*, and further suggests that ubiquitylation of H2B permits FACT to efficiently displace H2A/H2B histone dimers [14]. Here we have shown that the loss of Ctr9 or Paf1 *in vivo *is associated with delayed removal of histones from the *GAL1 *locus upon induction of transcription. This delay in histone displacement may contribute to the decrease in association and altered distribution of RNA pol II that we observed in Ctr9- or Paf1-deficient cells. Our findings may also explain the relative delay in induction of *GAL *transcripts in *ctr9Δ *and *paf1Δ *strains, relative to wild-type.

We have considered the possibility that a reduction in recruitment of RNA pol II, rather than an impairment of histone eviction, might be responsible for the delay in histone displacement that we observed. In support of our interpretation are two reports documenting recruitment of the Paf1 complex to promoter regions after, but not before, initiation of transcription. In mammalian cells the Paf1 complex is recruited to the *RARβ2 *promoter after recruitment of RNA pol II and FACT [14]. Moreover, RNA pol II binds to the yeast *ARG1 *promoter in the absence of the Paf1 complex; subsequent recruitment of the Paf1 complex is dependent on prior binding of the Spt4 transcription elongation factor and phosphorylation of RNA pol II at Ser5 [36]. Lastly, association of Rad6 and the SAGA complex with the *GAL1 *promoter occurs independently of H2B monoubiquitylation [31,32]. Taken together, these observations suggest that recruitment of RNA pol II to promoter regions occurs prior to and independent of the actions of Ctr9 or Paf1. Our results are consistent with a direct role for the Paf1 complex in promoting histone eviction but do not exclude the possibility that Paf1 and Ctr9 exert their effects through one or more additional components of the elongating RNA pol II complex.

It has been proposed that newly transcribed genes undergo a so-called "pioneer round" of transcription in which RNA pol II functions in concert with specialized machinery for chromatin modification [37]. Based on our work and earlier findings we suggest that upon initial induction of *GAL1*, Gal4, Rad6/Bre1, SAGA and RNA pol II, among other factors, are recruited and initiate transcription [31]. Subsequent association of the Paf1 complex with RNA pol II would promote monoubiquitylation of H2B at K123 by Rad6/Bre1 as the polymerase complex traverses the coding sequence [32]. We imagine that ubiquitylation of K123 could confer upon FACT and Asf1 the ability to evict histones from the DNA in addition to the established roles of FACT and Asf1 in histone displacement and redeposition. Our results suggest that in the absence of the Paf1 complex histones are inefficiently removed and transcriptional elongation is impaired.

While it has been suggested that the Paf1 complex does not affect transcriptional elongation from a *GAL1 *promoter [38], this conclusion was based on observations made at 2.5 hr after galactose induction. Because our data indicate that the effects of Ctr9 and Paf1 depletion on histone eviction are most evident early after induction, it is not surprising that differences in elongation rates were not observed at a relatively late time point. Our results suggest that the Paf1 complex exerts its effects on histone displacement during the initial stages of transcriptional activation, and that at later times after induction, histones can be removed even in the absence of the Paf1 complex.

The Paf1 complex and the H2B monoubiquitylation that it promotes are clearly not universally required for gene expression [39]. Unlike FACT, the genes encoding components of the Paf1 complex and Rad6/Bre1 are inessential [18,40,41]. The available data suggest that the Paf1 complex enhances transcriptional efficiency in those settings in which environmental changes demand rapid transcriptional responses, such as induction of metabolic enzymes or stress response genes. In this regard, it is particularly interesting that one salient phenotype of *paf1Δ *and *ctr9Δ *strains is sensitivity to a broad range of environmental stressors [42].

## Conclusion

In this communication we have shown that loss of either Paf1 or Ctr9 is associated with impaired induction of transcription at the *GAL1 *and *GAL10 *loci and altered recruitment and distribution of RNA pol II across the *GAL1 *locus. These effects are accompanied by delayed removal of histones from the *GAL1 *locus upon induction of transcription in Paf1- or Ctr9-deficient strains. Our findings suggest that the Paf1 complex and the histone modifications that it mediates increase the efficiency of transcriptional elongation by promoting destabilization of nucleosomes and histone removal.

## Methods

### Media, yeast strains and plasmids

Growth media were as described [43]. Glucose was the carbon source except where indicated. Yeast were maintained at 30°C unless noted. The strains used in this study were generated using standard techniques [44] and are listed in Table 1. Genetic manipulations were verified by Southern hybridization or PCR.

**Table 1:** Yeast strains used in this study

Strain	Genotype
HM158	*Mat a his3Δ1 leu2Δ0 ura3Δ0 ctr9::kanMX4 [pgCTR9M2]*
HM167	*Mat a his3Δ1 leu2Δ0 ura3Δ0 [pgCTR9M2]*
HM177	*Mat a his3Δ1 leu2Δ0 ura3Δ0*
HM198	*Mat a his3Δ1 leu2Δ0 ura3Δ0 CTR9-13 Myc::kanMX6*
HM199	*Mat a his3Δ1 leu2Δ0 ura3Δ0 PAF1-13 Myc::kanMX6*
HM200	*Mat a his3Δ1 leu2Δ0*
HM201	*Mat a his3Δ1 leu2Δ0 ctr9Δ::kanMX4*
HM202	*Mat a his3Δ1 leu2Δ0 paf1Δ::kanMX4*
YCB647^a^	*Mat a his3Δ200 leu2Δ1 lys2Δ202 trp1Δ63 ura3-52 leu2Δ::TRP1 ADH4::URA3-TEL*
HM180	*Mat a his3Δ200 leu2Δ1 lys2Δ202 trp1Δ63 ura3-52 leu2Δ::TRP1 ctr9Δ::kanMX4 ADH4::URA3-TEL*
HM205	*Mat a his3Δ200 leu2Δ1 lys2Δ202 trp1Δ63 ura3-52 leu2Δ::TRP1 paf1Δ::kanMX4 ADH4::URA3-TEL*
HM206	*Mat a his3Δ200 leu2Δ1 lys2Δ202 trp1Δ63 ura3-52 leu2Δ::TRP1 set1Δ::kanMX4 ADH4::URA3-TEL*
HM207	*Mat a his3Δ200 leu2Δ1 lys2Δ202 trp1Δ63 ura3-52 leu2Δ::TRP1 set2Δ::kanMX4 ADH4::URA3-TEL*
YCB761^a^	*Mat a ade2-101 his3Δ200 leu2Δ1 lys2-801 trp1Δ63 ura3-52 TEL-VR::ADE2*
HM182	*Mat a ade2-101 his3Δ200 leu2Δ1 lys2-801 trp1Δ63 ura3-52 ctr9Δ::kanMX4 TEL-VR::ADE2*
JS692^a^	*Mat a ade2-101 his3Δ200 leu2Δ1 lys2-801 trp1Δ63 ura3-52 npt1Δ::kanMX4 TEL-VR::ADE2*
JHY25^b^	*Mat a ade2::hisG his3Δ1 leu2Δ0 met15Δ0 ura3Δ0 can1Δ::MET15;AE – CAN1/ADE2/URA3 (XV)*
HM188	*Mat a ade2::hisG his3Δ1 leu2Δ0 met15Δ0 ura3Δ0 can1Δ::MET15 ctr9Δ::kanMX4;*
	*AE – CAN1/ADE2/URA3 (XV)*
HM189	*Mat a ade2::hisG his3Δ1 leu2Δ0 met15Δ0 ura3Δ0 can1Δ::MET15 bna1Δ::kanMX4;*
	*AE – CAN1/ADE2/URA3 (XV)*
HM190	*Mat a ade2::hisG his3Δ1 leu2Δ0 met15Δ0 ura3Δ0 can1Δ::MET15 npt1Δ::kanMX4;*
	*AE – CAN1/ADE2/URA3 (XV)*
JHY49^b^	*Mat a ade2::hisG his3Δ1 leu2Δ0 met15Δ0 ura3Δ0 can1Δ::MET15; TELO – CAN1/ADE2/URA3 (XV)*
HM191	*Mat a ade2::hisG his3Δ1 leu2Δ0 met15Δ0 ura3Δ0 can1Δ::MET15 ctr9Δ::kanMX4;*
	*TELO – CAN1/ADE2/URA3 (XV)*
HM192	*Mat a ade2::hisG his3Δ1 leu2Δ0 met15Δ0 ura3Δ0 can1Δ::MET15 bna1Δ::kanMX4;*
	*TELO – CAN1/ADE2/URA3 (XV)*
HM193	*Mat a ade2::hisG his3Δ1 leu2Δ0 met15Δ0 ura3Δ0 can1Δ::MET15 npt1Δ::kanMX4;*
	*TELO – CAN1/ADE2/URA3 (XV)*

^a^Ref 28.

^b^Ref 27.

To construct a plasmid for expression of Ctr9, PCR was used to generate a DNA fragment encoding full-length *CTR9*, tagged at the carboxyl terminus with two c-myc epitopes; this was transferred to the p415-GAL1 expression vector to generate pgCTR9M2 [45].

### Measurement of intracellular NAD^+^

Strains were grown in SC -leu + 2% galactose to an O.D. of 1.0. An aliquot of each culture was removed for the 0 hr time point. The remaining cells were transferred to SC -leu + 2% glucose. At 4, 8 and 12 hr, intracellular NAD^+ ^were assayed as described [46].

### Chromatin immunoprecipitation

Strains expressing endogenous, c-myc-tagged Ctr9 or Paf1, and the untagged parental strain, were grown in YPD. ChIP was performed as described [47]; cultures were treated with 1% formaldehyde for 30 min. DNA fragments were immunoprecipitated with the 9E10 anti-c-myc antibody. Association of c-myc-tagged Ctr9 and Paf1 with specific DNA segments was monitored by PCR amplification. PCR reactions (25 μl) contained 1/50 of the precipitated DNA, 0.25 mM of each dNTP, 12.5 pmol of each primer, 0.75 μCi [^32^P]-dCTP and 1.6 U Taq polymerase (Roche). Reactions were incubated for 4 min at 95°C, followed by 22 amplification cycles (40 sec at 95°C, 40 sec at 52°C, 45 sec at 72°C) and a 5 min extension step at 72°C.

The following gene segments (defined relative to translation start sites) were assayed for the presence of Ctr9 and Paf1: *ACT1 *(+1516 to +1737 bp), *ADH3 *(+569 to +818 bp), *BNA1 *(+201 to +522 bp), *BNA4 *(+1889 to +2225 bp), *CWP1 *(+1401 to +1719 bp), *EST3 *(+268 to +534 bp), *RPT5 *(+758 to +994 bp), *SEC59 *(+918 to +1206 bp) and *TAF10 *(+8 to +243 bp). A fragment from the *ARN1 *promoter (-260 to -83 bp) was used as a reference.

For ChIP assays after galactose induction, cells were grown at 24°C to an O.D. of 0.8 – 0.9 in YEP with 2% raffinose. Cultures were induced with 2% galactose and samples were removed at 0, 5, 10, 20, 40, 60 and 90 min post-induction. Chromatin was prepared as described above. DNA was precipitated using the anti-Rpb3 antibody 1Y26 (Neoclone) and an anti-histone H3 antibody (Abcam). Specific DNA was detected by real time PCR (ABI). Sequences from 5' (+17 to +80), middle (+633 to +696) and 3' (+1374 to +1440) regions of *GAL1 *were detected with SYBR Green (ABI) using standard amplification conditions (40 cycles; 95°C for 15 s, 60°C for 1 min). A portion of the *FBA1 *gene and an untranscribed, intergenic region (between *NRG1 *and *HEM13*) were amplified as controls. Precipitation of each region of *GAL1 *was normalized to that of the *FBA1 *(RNA pol II) or intergenic (H3) controls and expressed relative to the amount of association prior to galactose induction.

### Assays of telomeric silencing

*URA3 *strains were grown on SC plates. Serial dilutions were spotted onto SC, SC -ura, and 5 FOA (0.1%) plates. To assay *ADE2 *silencing, strains were grown on SC plates. About 200 cells from each strain were plated on SC containing limiting adenine.

### *GAL *induction assays

Strains were grown at 30°C to O.D. 0.4 – 0.5 in YEP supplemented with 2% raffinose. An aliquot of each culture (10 O.D. units) was removed and frozen at -80°C. To the remainder of the cultures, galactose was added to 2%. At 5, 15, 30, 45 and 60 min after addition of galactose, cells (10 O.D. units) were sampled. A second induction assay was repeated as above with minor changes. Cells were grown at 24°C to an O.D. of 0.6. Samples were removed at 15, 30, 45 and 60 min after addition of galactose. *GAL1, GAL10 *and *ACT1 *transcripts were assayed by northern hybridization.

### Micrococcal nuclease digestion

Digest of genomic chromatin with micrococcal nuclease was performed as described with the minor modifications indicated below [48]. Yeast strains were grown at 24°C to an O.D. of 0.8 – 0.9 in YEP supplemented with 2% raffinose. Cultures were induced with 2% galactose for 0, 10 and 90 min. The preincubation solution consisted of 0.7 M β-mercaptoethanol, 10 mM EDTA (pH 8.0). Spheroplasts were prepared by addition of 3.5 mg of zymolase-100T (Seikagaku) per g of cells and the suspension was shaken at 37°C for 10 min. Samples were spun at 48,000 × g at 4°C for 30 min. Samples were digested with 0, 7, 15, 50 or 100 U micrococcal nuclease (USB). After deproteinization, samples (20 μg) were resolved on 1.1% agarose and bulk DNA was detected with ethidium bromide. *GAL1 *DNA was detected by Southern hybridization.

## Authors' contributions

HAM conceived and executed all experiments and drafted the manuscript. SD helped to conceive and design the study, analyzed data and drafted the manuscript. Both authors have read and approved the final manuscript.
